# The growth-promoting and disease-suppressing mechanisms of *Trichoderma* inoculation on peanut seedlings

**DOI:** 10.3389/fpls.2024.1414193

**Published:** 2024-06-25

**Authors:** Xingqiang Wang, Zhongjuan Zhao, Hongmei Li, Yanli Wei, Jindong Hu, Han Yang, Yi Zhou, Jishun Li

**Affiliations:** ^1^ Shandong Provincial Key Laboratory of Applied Microbiology, Ecology Institute of Qilu University of Technology (Shandong Academy of Sciences), Jinan, China; ^2^ China–Australia Joint Laboratory for Soil Ecological Health and Remediation, Ecology Institute of Qilu University of Technology (Shandong Academy of Sciences), Jinan, China; ^3^ School of Agriculture, Food and Wine, The University of Adelaide, Urrbrae, SA, Australia

**Keywords:** soil health, microbiome, omics, ACCd, fungal, legume

## Abstract

*Trichoderma* spp. is known for its ability to enhance plant growth and suppress disease, but the mechanisms for its interaction with host plants and pathogens remain unclear. This study investigated the transcriptomics and metabolomics of peanut plants (*Arachis hypogaea* L.) inoculated with *Trichoderma harzianum* QT20045, in the absence and presence of the stem rot pathogen *Sclerotium rolfsii* JN3011. Under the condition without pathogen stress, the peanut seedlings inoculated with QT20045 showed improved root length and plant weight, increased indole acetic acid (IAA) production, and reduced ethylene level, with more active 1-aminocyclopropane-1-carboxylate acid (ACC) synthase (ACS) and ACC oxidase (ACO), compared with the non-inoculated control. Under the pathogen stress, the biocontrol efficacy of QT20045 against *S. rolfsii* was 78.51%, with a similar effect on plant growth, and IAA and ethylene metabolisms to the condition with no biotic stress. Transcriptomic analysis of peanut root revealed that *Trichoderma* inoculation upregulated the expression of certain genes in the IAA family but downregulated the genes in the ACO family (*AhACO1* and *AhACO*) and ACS family (*AhACS3* and *AhACS1*) consistently in the absence and presence of pathogens. During pathogen stress, QT20045 inoculation leads to the downregulation of the genes in the pectinesterase family to keep the host plant’s cell wall stable, along with upregulation of the *AhSUMM2* gene to activate plant defense responses. *In vitro* antagonistic test confirmed that QT20045 suppressed *S. rolfsii* growth through mechanisms of mycelial entanglement, papillary protrusions, and decomposition. Our findings highlight that *Trichoderma* inoculation is a promising tool for sustainable agriculture, offering multiple benefits from pathogen control to enhanced plant growth and soil health.

## 1 Introduction

Stem rot disease in peanuts (*Arachis hypogaea* L.), caused by a soil-borne necrotrophic fungus *Sclerotium rolfsii* Sacc, is prevalent in the world’s major peanut-producing regions (Subedi et al., 2024), including China, India, US, and Argentina (Bot et al., 2011), and causes yield loss of up to 80% (Yan et al., 2021). *S. rolfsii* secretes an exceptionally large quantity of polygalacturonases, cutinase, and cellulase enzymes that break down the primary and secondary cell wall components of host cells (Ayyandurai et al., 2023). The disease shows up as the yellowing and dropping of the lower branches on the main stem as well as poor root growth, stem rot, and early death (Aydoğdu, 2023 ). This pathogen can also form sclerotia in the soil or in host plant debris for a long period, leading to continued infections (Schmid et al., 2010); hence, agricultural practices such as crop rotation, deep plowing, and residue removal were ineffective to control the stem rot disease (Jia et al., 2023). Chemical control including the use of fungicides such as tebuconazole, difenoconazole, and chlorothalonil (Cilliers et al., 2003) serves as the primary method to control this disease, but the extensive use of pesticides leads to soil pollution and pathogen resistance—for example, the United States Environmental Protection Agency has labeled tebuconazole as a potential carcinogen for humans (Cui et al., 2018). Tebuconazole has been observed to move from soil into aquatic systems via runoff and frequently detected at 0.010–0.115 μg/L in US streams (Bradley et al., 2017). Therefore, using novel biocontrol agents with effective antagonistic properties is considered as a sustainable approach for the management of peanut stem rot disease (Whipps, 2004).

*Trichoderma* spp. has been widely used as a beneficial fungus, which exhibits a strong capacity to promote host plant growth and suppress disease infection (López-Bucio et al., 2015; Zhou et al., 2020; Liu et al., 2022). *Trichoderma* strains stimulate plant growth, including seed germination, root elongation, flowering time, and fruit formation by modulating the plant hormone metabolism and signaling (Viterbo et al., 2010). Firstly, auxin-related compounds produced by *T. virens* played a great role in modulating the root architecture and activation of auxin-regulated gene expression in *Arabidopsis* plants (Contreras-Cornejo et al., 2009). In addition, the presence of the 1-aminocyclopropane-1-carboxylate acid (ACC) deaminase gene in *Trichoderma* species genomes leads to the regulation of ethylene levels in plants, and ethylene biosynthesis acts as a key regulator between environmental shifts and plant adaptive responses (Pieterse et al., 2012)—for example, the study on wheat seedling under salt stress revealed that the ACC deaminase activity from *T. longibrachiatum* strain increased the plant IAA concentration by 11%, decreased the ACC content by 22%, and reduced the ethylene level by 12% (Zhang et al., 2019).

Under biotic stress, *Trichoderma* is also an important biocontrol agent against pathogenic fungus. The antagonistic mechanism of *Trichoderma* against pathogens mainly includes mycoparasitism and inhibitory effect from secreting the antimicrobial compounds. In the mycoparasitism process, *Trichoderma* firstly recognizes the oligochitins in the pathogen’s cell wall and then grows toward the pathogen by chemotropism. Upon physical contact, the hyphae of *Trichoderma* wrap around those of the pathogen, triggering the release of enzymes that degrade the cell walls, such as chitinases and β-1,3-glucanases (Sood et al., 2020). Meanwhile, *Trichoderma* can also release the volatile organic compounds (VOCs) acting as antagonistic agents (Kanchiswamy et al., 2015). The study by which *Trichoderma* spp. (*T. longibrachiatum* and *T. asperellum*) was co-cultured with *S. rolfsii* found that the specific antimicrobial VOCs of isolongifolan-7-ol and transsesquisabinene hydrate showed antifungal activity against *S. rolfsii* (Ayyandurai et al., 2023). However, there is insufficient understanding regarding how *Trichoderma* influences plant hormone metabolism during its interaction with pathogens.

*Trichoderma* spp. not only directly interacts with phytopathogens but also reprograms the gene expression of the host plants, thereby stimulating defense mechanisms to suppress the pathogen (Lorito et al., 2010; Druzhinina et al., 2011; Ray et al., 2016). The plant response induced by *Trichoderma* inoculation was stronger than that of immunity triggered by pathogens (Cornejo et al., 2011). Even during the early stages of plant growth, there exists a substantial communication between the host plant and *Trichoderma*. Plant seedlings subjected to *Trichoderma* treatment, either with crude mycelium or purified proteins such as chitinase and cellulase, displayed various cellular alterations linked to defense mechanisms (Sriram et al., 2009), including rapid ion fluxes, the production of reactive oxygen compounds, the accumulation of phytoalexins, and the synthesis of pathogenesis-related proteins (Abbas et al., 2022).

For a legume plant which is able to form symbiosis with the rhizobia, there is an interaction between disease infection and symbiosis on the transcriptional response of the host plant, e.g., metabolism and signaling pathways (Maheshwari et al., 2021; Zhou et al., 2022). However, the impact of *Trichoderma* inoculation on the metabolism of legume plants in the presence and the absence of pathogen infections remained largely unknown. This study focuses on the hormone (IAA and ethylene) metabolism pathways of peanut host responding to the *T. harzianum* inoculation with and without the stress of *S. rolfsii* pathogen. The research objective is to evaluate the mechanisms of *T. harzianum* inoculation that promoted peanut growth under non-stress conditions and the mechanisms by which *T. harzianum* inoculation suppressed the *S. rolfsii* pathogen and modulated host plant metabolisms to resist disease.

## 2 Materials and methods

*Trichoderma harzianum* strain QT20045 was used in this study. This strain was obtained from our *Trichoderma* Resource Bank in the Ecological Institute of Shandong Academy of Sciences, NCBI accession no. MH284507 (Hu et al., 2020). *Sclerotium rolfsii* JN3011 was isolated from diseased peanuts with stem rot collected from the peanut fields in Jinan City, China. The CAS number and brand of the key chemicals used in this article are listed in **Supplementary Table S3**.

### 2.1 *In vitro* antagonistic test of *T. harzianum* QT20045 against *S. rolfsii*

The antagonistic effect of *T. harzianum* QT20045 against *S. rolfsii* was evaluated under *in vitro* condition using the dual culture method. Three treatments including *T. harzianum* QT20045 (+T-S), *S. rolfsii* (-T+S), and QT20045 and *S. rolfsii* dual culture (+T+S) were studied as a completely randomized design with three experimental replicates. *T. harzianum* QT20045 and *S. rolfsii* were purely cultured in potato dextrose agar (PDA) medium at 25°C for 4 days. The mycelium disc from each culture was co-inoculated on the edges of the PDA plate, which was then incubated at 25°C. The mycelium disc of individual fungus was also inoculated in separate PDA plates as the control. The percentage of inhibition was calculated as the relative increase for the diameter of pathogenic fungus without *Trichoderma* to the diameter of pathogenic fungus in the treatment with *Trichoderma*.

The conidia suspension of *T. harzianum* QT20045 was obtained by spores from the individual culture and dual culture with *S. rolfsii*, respectively. The procedure began by pouring 3 mL of sterilized distilled water onto the dishes, and the suspension was collected. The concentration of conidia was evaluated using a hemocytometer and then adjusted to reach a concentration of 1 × 10^8^ CFU/mL. The *Trichoderma* suspension was used for the analysis of IAA production and ACC deaminase activity according to the method from Penrose and Glick (2010), respectively. The concentration of IAA was determined by colorimetric method with OD_530_ and calculated by comparison with the IAA standard curve. The total protein concentration in extract was determined by a dye-binding method using bovine serum albumin as the protein standard (Kruger, 2009). The activity of ACC deaminase was quantitatively evaluated by measuring the amount of α-ketobutyrate produced, expressed as µmol α-ketobutyrate produced per milligram of protein per hour.

### 2.2 Biocontrol efficacy of *T. harzianum* QT20045 against *S. rolfsii* under greenhouse conditions

The biocontrol efficacy of QT20045 against *S. rolfsii* was *in vivo* evaluated in peanut plants growing under greenhouse conditions. The experiment was carried out with four treatments and five replicates using a completely randomized design. Four treatments included inoculating with *T. harzianum* QT20045 and *S. rolfsii* and the non-inoculated controls (+T-S, -T-S, +T+S, and -T+S). The peanut seeds (cultivar “Luhua 19” provided by Shandong Luhua Agricultural Science and Technology Promotion Co. Ltd.) were sterilized in 70% ethanol for 2 min and 2.0% NaClO for 2 min and then washed with sterile water three times. The disinfected seeds were soaked in sterilized water or QT20045 conidia suspension (1 × 10^8^ CFU/mL) for 1 h and then incubated at 25°C for 2 days. The germinated seeds were planted in pots (115-mm high × 92-mm wide × 114-mm long) containing 1 kg of sterilized soil with three seedlings in each pot and cultivated in the greenhouse at 28°C in the daytime and 18°C at night. The pathogenic fungal solution was prepared by culturing in PDB at 180 rpm for 7 days, and 20 mL solution was watered to the peanut seedlings’ root. The plants were harvested at 20 days after pathogen infection when the vegetation growth reached the maximum. Plant height was measured, and roots were washed with sterilized water for the further analysis. The disease incidence (percentage of plants showing disease symptoms) of peanut plants was assessed, and the severity of stem rot disease was scored visually on a scale of 0–4 based on the percentage of affected leaves and twigs: 0 indicated no symptoms, 1 represented 1%–33% affected, 2 indicated 34%–66% affected, 3 represented 67%–100% affected, and 4 indicated a dead plant (Carrero-Carron et al., 2016). The disease index (DI) of each treatment was calculated using the Townsend–Heuberger formula (Townsend and Heubergeb, 1943) as below:


$$DI\;\left(\%\right)=\frac{\sum \left(r\times Nr\right)}{4\times N}\times 100\%$$

where *r* is the rating value (0–4), *Nr* is the number of disease plants with a rating of *r*, and *N* is the total number of peanut plants.

The control efficacy was calculated using Abbott’s formula (Abbott, 1925): the relative increase for the disease severity without QT20045 (infected with *S. rolfsii*) to the disease severity in QT20045 treatment (infected with *S. rolfsii*).

### 2.3 Hormone metabolism analysis of peanut seedlings under the inoculation of *T. harzianum* QT20045 and pathogen *S. rolfsii*

The endogenous IAA was extracted from plant roots and purified using the modified method from Alemneh et al. (2021). Root samples (1 g) were frozen immediately after harvest and then homogenized with a mortar and pestle using 80% methanol for IAA extraction. High-performance liquid chromatography (HPLC; model Agilent 1100, Agilent Technologies, Waldbronn, Germany), utilizing a C18 column with a 5-µm particle size and an ultraviolet (UV) detector with a wavelength of 221 nm, was used for IAA detection. In the mobile phase, methanol/acetonitrile/acetic acid (0.1%), in a 60:5:35 ratio, was applied at a flow rate of 0.8 mL/min and column temperature of 30°C. The ethylene synthesis in peanut seedlings was measured following the method of Yamauchi et al. (2014). Fresh roots (1 g) were placed into a 20-mL airtight glass bottle with 10 mL water to maintain root moisture. After incubating, 1 mL gas was collected from the bottle and analyzed through gas chromatography–mass spectrometry (GC–MS, Agilent 7890B; Agilent, Foster City, CA, USA). The concentration of ethylene in a standard sample was determined to make the standard curve. Ethylene production was expressed in µmol per gram of root fresh weight per hour.

The activity of ACS was detected by modifying the method of Kato et al. (2000). Peanut root samples (0.3 g) were ground in liquid nitrogen, then transferred to a 2-mL centrifuge tube with 1.5 mL of a mixed solution containing 100 mM HEPPS-KOH extraction buffer (pH 8.5), 10 mM β-sulfhydryl ethanol, 10 µM pyridoxal phosphate, and 1 mM EDTA, and centrifuged at 12,000 rpm and 4°C for 20 min. The supernatant, as the crude enzyme extract, was determined by a dye-binding method using bovine serum albumin as the protein standard (Kruger, 2009). After that, 0.7 mL of the supernatant was transferred into a headspace vial with 2 mL of a mixed solution containing 50 mM HEPPS-KOH reaction buffer (pH 8.5), 250 µM S-adenosyl-L-methionine (SAM), and 10 µM pyridoxal phosphate and sealed with a silicone stopper. After incubation, 1 mL of gas was collected from the headspace vial to quantify the ethylene content using GC–MS. ACS activity was determined as the amount of ethylene converted from SAM during the reaction period and expressed as µmoles ACC per gram protein per hour.

The activity of ACC oxidase (ACO) was detected following the method described by Nguyen et al. (2023). Firstly, 0.3 g of peanut roots was ground in liquid nitrogen, then transferred to a headspace vial containing 4 mL of a solution containing 100 mM MOPS buffer (pH 7.2), 1 mM ACC, 1 mM ascorbic acid, and 200 µM FeSO_4_. To provide the CO_2_ for reaction, 50 mL of 30 mM NaHCO_3_ was injected into the bottle. The ethylene content in the gas was analyzed using GC–MS. The ACO activity was determined by quantifying the ethylene converted from ACC during the reaction, expressed as µmoles ACC per gram of protein per hour.

### 2.4 Transcriptome analysis of peanut seedlings under the inoculation of *T. harzianum* QT20045 and pathogen *S. rolfsii*

Total RNA was isolated from 0.1 g root samples (ground into powder in liquid nitrogen) using RNA prep Pure Plant Plus Kit (Norgen Biotek, Canada) according to the manufacturer’s instructions. RNA concentration and quality were determined using a NanoPhotometer spectrophotometer (Thermo Fisher Scientific, USA). mRNA was isolated from total RNA using magnetic beads attached to poly-T oligos. Fragmentation was conducted at high temperature with divalent cations in NEBNext’s First Strand Synthesis Reaction Buffer (5X). Random hexamer primers and M-MuLV Reverse Transcriptase (RNase H-) were employed to synthesize first-strand cDNA. Subsequently, second-strand cDNA synthesis was carried out using DNA Polymerase I and RNase H. The sequencing libraries were generated using NEBNext^®^ UltraTM RNA Library Prep Kit for Illumina^®^ (Illumina, USA) following the manufacturer’s recommendations. Sequencing was performed using the Illumina Novaseq PE150 platform. The raw data were quality-controlled using Fastp (version 0.23.1) (Chen et al., 2018) to produce clean data. The reference genome was built using Hisat2 (version 2.2.1) (Kim et al., 2015), and paired-end clean reads were aligned to the reference genome. Validated data were compared to the assembled transcriptome sequence, and FeatureCounts (version 2.0.1) (Liao et al., 2013) was used to determine the expression levels. DESeq2 (version 1.26.0) (Love et al., 2014) in R package was used for differential gene expression analysis, and genes with log fold change ≥1 and adjusted *P*-value<0.05 (the resulting *P*-values were adjusted using Benjamini and Hochberg’s approach to control the false discovery rate) were considered significantly differentially expressed genes (DEGs) (Jung et al., 2011). We used ClusterProfiler (version 3.4.4) (Yu et al., 2012) in R package for GO enrichment analysis and Kyoto Encyclopedia of Genes and Genomes (KEGG) pathway enrichment analysis (Gao et al., 2024).

We selected two of the most representative genes, meeting the criteria of log fold change >1 and adjusted *P*-value<0.05, from each of the IAA, ACO, and ACS families and one gene from each of the PR1, PME, and DFR families related to plant disease resistance. In total, the expression of nine selected DEGs was analyzed by RT-qPCR to validate the RNA-seq data. Primers with lengths that ranged from 200 to 300 bp were designed using Primer 5.0 software (Bai and Wong, 2004) (the primer sequences are listed in **Supplementary Table S1**). First-strand cDNA was reverse-transcribed from the extracted total RNA of the peanut roots for RNA-seq using the Evo M-MLV RT Premix kit (Promega, USA) according to the manufacturer’s instructions. RT-qPCR was performed using a CFX96 optical real-time detection system (Bio-Rad Laboratories Inc, USA). The 20-µL reactions contained 10 µL 2xPower SYBR^®^ Green PCR Master Mix (Thermo Fisher Scientific, USA), 0.4 µL each of 10 µM forward and reverse primers, 2.0 µL cDNA samples, and 7.2 µL sterile and DNA-free water. The PCR program was as follows: pre-denaturation at 95°C for 30 s, denaturation at 95°C for 5 s, annealing at 54°C for 30 s, and extension at 72°C for 20 s, for a total of 40 cycles. Amplicon dissociation curves were recorded after cycle 40 by heating from 60°C to 95°C with a ramp speed of 1.0°C per min. All reactions were performed with three technical replicates for each of the biological replicates. Actin-7 was used as the internal reference gene, and the relative expression was calculated using the method from Schmittgen and Livak (2008).

### 2.5 Statistical analyses

After confirming the normal distribution of the measured variables, one-way analysis of variance (ANOVA) for a completely randomized design, with three replicates for the variables in the *in vitro* experiment and five replicates for the variables in the pot experiment, was performed using SPSS (version 26.0), and Duncan’s multiple-range test was applied to compare the different treatments at *p*<0.05.

## 3 Results

### 3.1 Antagonistic activity of *T. harzianum* QT20045 against *S. rolfsii*

**Figure 1:**
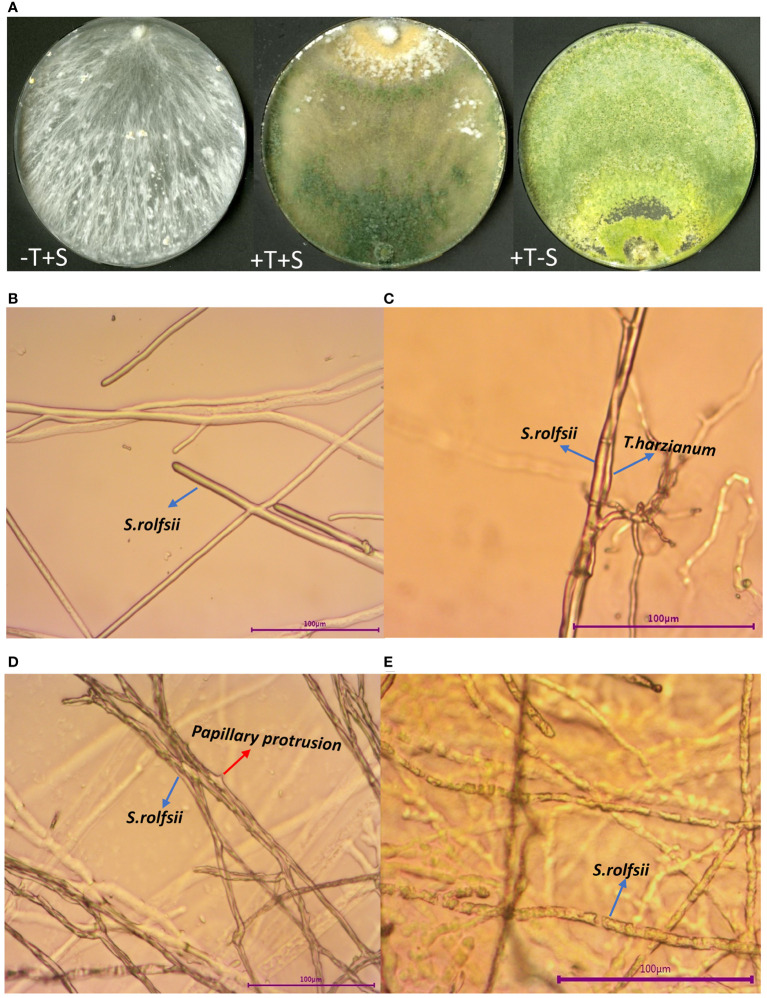
*Trichoderma harzianum* QT20045 inhibits the mycelial growth of *Sclerotium rolfsii*. Two fungi were cultured in potato dextrose agar medium **(A)** with the treatments of -T+S (*S. rolfsii* individually), +T+S (*S. rolfsii* dual-cultured with *T. harzianum* QT20045), and +T-S (*T. harzianum* QT20045 individually). Morphological characteristics of *S. rolfsii* mycelia cultured without *T. harzianum* QT20045 **(B)** and interacted with *T. harzianum* QT20045 including entanglement **(C)**, papillary protrusions **(D)**, and decomposition **(E)**.

*In vitro* test on PDA medium showed that *T. harzianum* QT20045 suppressed the growth of *S. rolfsii* (**Figure 1A**), and the percentage of inhibition was 97.60% ± 2.92%. Morphological changes of the *S. rolfsii* colony, when cultured with *T. harzianum* QT20045, were observed using an optical microscope (**Figure 1B**). The interactions between *S. rolfsii* and *T. harzianum* QT20045 include mycelial entanglement (**Figure 1C**), papillary protrusions (**Figure 1D**), and decomposition (**Figure 1E**).

**Figure 2:**
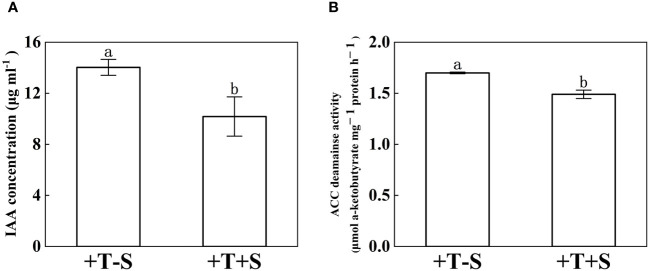
Indole acetic acid concentration **(A)** and 1-aminocyclopropane-1-carboxylate acid-deaminase activity **(B)** of *T. harzianum* QT20045 under individual culturing (+T-S) and dual-culturing with *S. rolfsii* (+T+S). The bars represent the average of the biological replicates (*n* = 3) with standard errors. Different letters denote significant differences at *p*< 0.05 by Duncan’s new multiple-range test.

The IAA concentration and ACC deaminase activity of *T. harzianum* QT20045 were measured under individual culture and dual culture with *S. rolfsii* (**Figure 2**). Compared with the individual culture, *T. harzianum* QT20045 under the dual culture decreased its IAA production by 27.46% (**Figure 2A**, *p*< 0.05) and ACC deaminase activity by 12.34% (**Figure 2B**, *p*< 0.05).

### 3.2 Effect of *T. harzianum* QT20045 on the growth and stem rot disease control of peanut under greenhouse conditions

**Figure 3:**
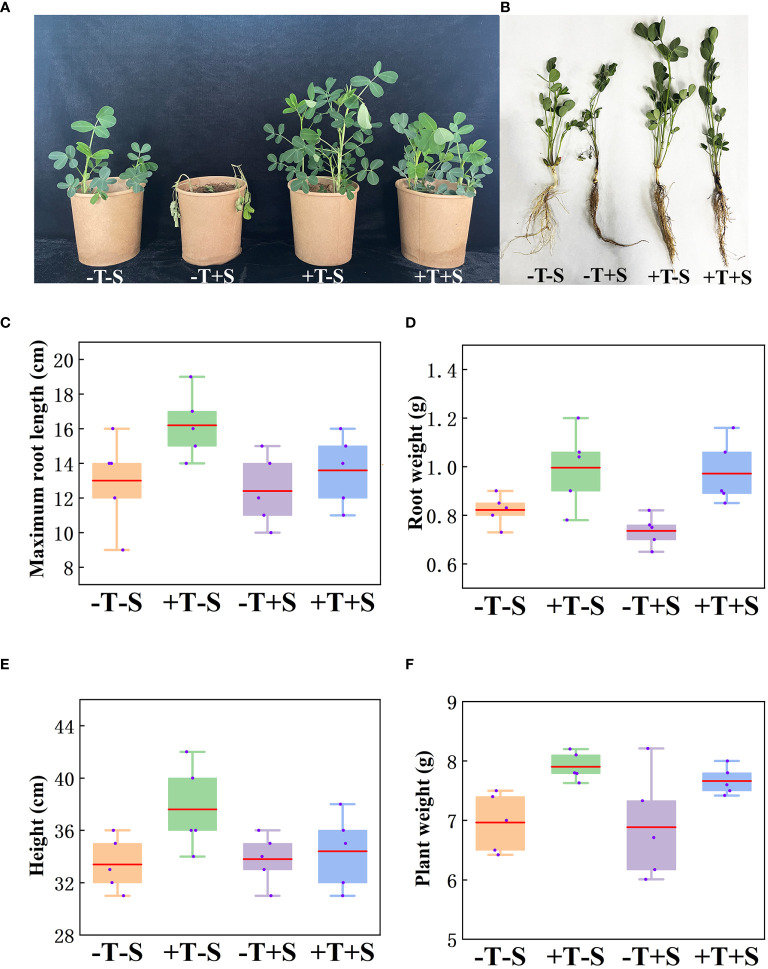
Growth-promoting and biocontrol effect of *T. harzianum* QT20045 on peanut seedlings after 20 days of growth, including the observed plant shoots **(A)** and roots **(B)** and the measured maximum root length **(C)**, root weight **(D)**, plant height **(E)**, and plant weight **(F)**. The experimental treatments included the combinations of inoculation with *T. harzianum* QT20045 (+T and -T) and the pathogen *S. rolfsii* (+S and -S).

The biocontrol efficacy of *T. harzianum* QT20045 against *S. rolfsii* was investigated under pot condition in the soil (**Figure 3A**). The result showed that the peanut seedlings treated with QT20045 displayed promoted growth when no pathogen was added and less disease infection when the pathogen was inoculated (**Figure 3**, **Supplementary Table S1**).

Under no pathogen inoculation, compared with the control (-T-S), the peanut plants treated with QT20045 (+T-S) displayed an obvious increase in maximum root length by 24.62%, root weight by 21.17%, plant weight by 13.50%, and plant height by 12.57% (**Figures 3C–F**). When a pathogen was present, compared with the control (-T+S), the peanut plants treated with QT20045 (+T+S) also significantly improved the maximum root length by 9.68%, root weight by 29.44%, plant weight by 11.30%, and plant height by 5.52% (**Figures 3C–F**). The disease incidence and disease index of peanut plants infected with *S. rolfsii* reached 61.27% and 36.19%, respectively, and *T. harzianum* QT20045 remarkably reduced them, resulting in the control efficiency of 78.51% (**Supplementary Table S1**).

**Figure 4:**
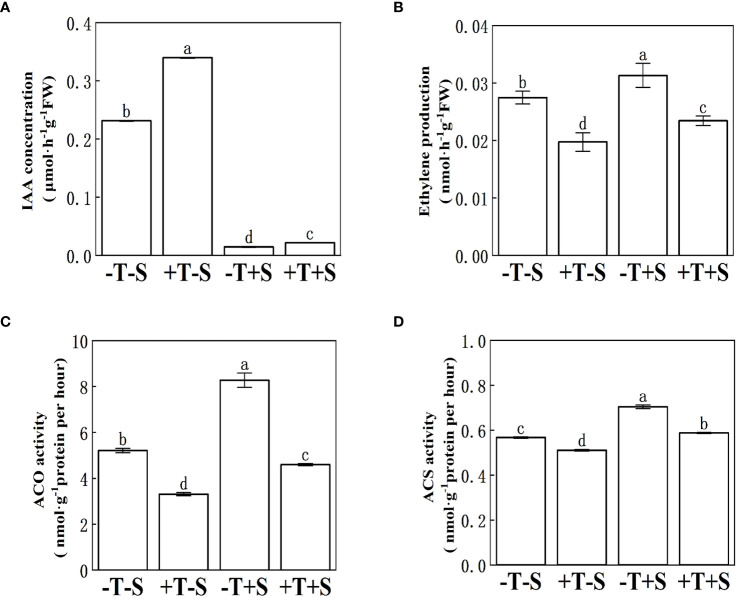
Effect of *T. harzianum* QT20045 inoculation on indole acetic acid content **(A)**, ethylene production **(B)**, 1-aminocyclopropane-1-carboxylate acid oxidase activity **(C)**, and 1-aminocyclopropane-1-carboxylate acid synthase activity **(D)** in the roots of peanut seedlings. The experimental treatments included the combinations of inoculation with *T. harzianum* QT20045 (+T and -T) and the pathogen *S. rolfsii* (+S and -S). The bars represent the average of the biological replicates (*n* = 5) with standard errors. Different letters denote significant difference at *p*< 0.05 by Duncan’s new multiple-range test.

Under no pathogen inoculation, the peanut seedlings treated with *T. harzianum* QT20045 (+T-S) produced more IAA (**Figure 4A**) and less ethylene (**Figure 4B**) compared with the non-*Trichoderma*-inoculated control (-T-S). Under the biotic stress of *S. rolfsii*, the application of *T. harzianum* QT20045 (+T+S) also increased the IAA production by 48.08% (**Figure 4A**) and decreased ethylene release by 25.18% (**Figure 4B**) in the roots of peanut plants compared with the control (-T+S).

The effect of *T. harzianum* QT20045 inoculation on the ACS activity and ACO activity in the roots of peanut seedlings (**Figures 4C, D**) was consistent with its influence in ethylene release (**Figure 4B**). Compared with the non-inoculated control, *T. harzianum* QT20045 significantly reduced the ACS activity and ACO activity of the peanut plants when the pathogen was not (**Figures 4C, D**).

### 3.3 Gene expression profiles of peanut seedlings under the inoculation of *T. harzianum* QT20045 and pathogen *S. rolfsii*

In order to analyze the genes related to the response of peanut plants to *T. harzianum* QT20045 (+T and -T) and *S. rolfsii* (+S and -S), RNA-seq was carried out using the total RNA of peanut seedlings. In total, 42.7, 43.1, 42.2, and 40.7 million reads with a length of 143–150 bp were obtained from -T-S, +T-S, -T+S, and +T+S treatments, respectively.

**Figure 5:**
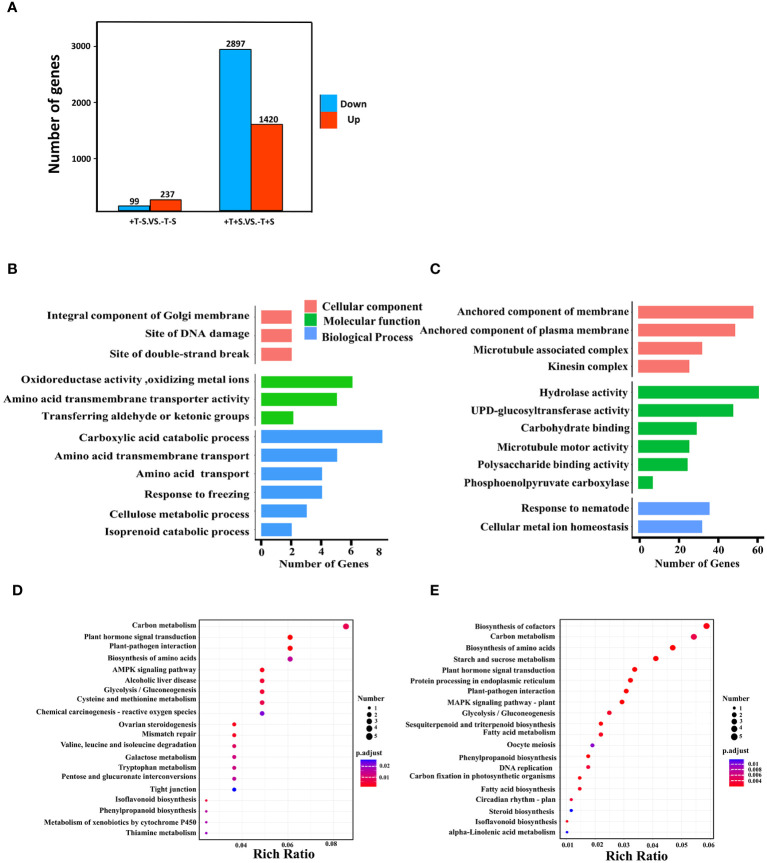
Effect of *T. harzianum* QT20045 inoculation (+T and -T) on root transcriptomes of peanut seedling in the absence and presence of the pathogen *S. rolfsii* (+S and -S) with focus on two comparisons +T-S vs. -T-S and +T+S vs. -T+S. Number of upregulated and downregulated differentially expressed genes (DEGs) in two comparisons **(A)**. GO terms significantly enriched in biological processes, cellular components, and molecular functions for +T-S vs. -T-S comparison **(B)** and +T+S vs. -T+S comparison **(C)**. KEGG pathway enrichment scatter map for +T-S vs. -T-S comparison **(D)** and +T+S vs. -T+S comparison **(E)**. Rich ratio is the ratio of the DEGs number to all the gene numbers annotated in the pathway.

To focus on the effect of *T. harzianum* QT20045 on the transcriptomes of peanut plants, we selected two crucial comparison groups: +T-S vs. -T-S and +T+S vs. -T+S. Gene expression analysis showed that 336 DEGs were identified in +T-S vs. -T-S comparison, including 237 DEGs being upregulated and 99 DEGs being downregulated, and 4,317 DEGs were identified in +T+S vs. -T+S comparison, including 1,420 up-regulated DEGs and 2,897 downregulated DEGs (**Figure 5A**).

GO analysis revealed that all DEGs fell into three functional categories: biological process, cellular component, and molecular function. In the comparison of +T-S vs. -T-S, DEGs were significantly enriched in the functional groups of biological processes (e.g., carboxylic acid catabolism and amino acid transmembrane transport), molecular functions (e.g., oxidoreductase activity and amino acid transmembrane transporter activity), and cellular components (e.g., integral components of the Golgi membrane, DNA damage sites, and double-strand break sites; **Figure 5B**). In the +T+S vs. -T+S comparison, DEGs were significantly enriched in the pathways of biological processes including responses to nematodes and cellular metal ion homeostasis, molecular functions such as hydrolase activity and UDP-glucosyltransferase activity, and cellular components related to anchored membrane (**Figure 5C**).

To further explore DEG functions, we conducted a KEGG enrichment analysis. We identified 284 DEGs across 178 pathways from the +T-S vs. -T-S comparison. The top pathways included carbon metabolism (seven DEGs; two upregulated and five downregulated), plant hormone signal transduction (five DEGs; four upregulated and one downregulated), and plant–pathogen interaction (five DEGs; three up regulated and two down regulated) (**Figure 5D**). For the +T+S vs. -T+S comparison, 2,200 DEGs distributed across 378 pathways were identified, including the key pathways for biosynthesis of cofactors (40 DEGs; seven upregulated and 33 downregulated), carbon metabolism (37 DEGs; six upregulated and 31 downregulated), and biosynthesis of amino acids (32 DEGs; five upregulated and 27 downregulated) (**Figure 5E**).

**Figure 6:**
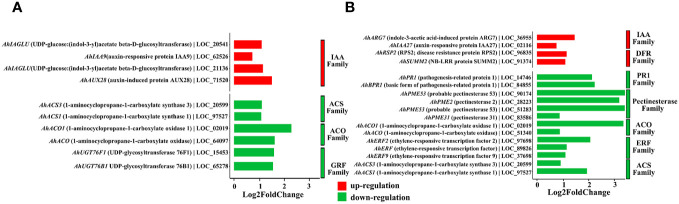
Differentially expressed genes of peanut seedling induced by *T. harzianum* QT20045 inoculation in the absence **(A)** (T-S vs. -T-S) and presence **(B)** (+T+S vs. -T+S) of the pathogen *S. rolfsii*.

When annotating the DEGs, we found that most of them were enriched in functions related to the modulation of plant growth and resistance to plant diseases. For the effect of QT20045 on plant growth in the absence of the pathogen, the IAA-related family (including the genes *AhIAGLU*: LOC_20541, *AhIAA9*: LOC_62526, *AhIAGLU*: LOC_21136, *AhAUX28*: LOC_71520) was significantly upregulated by *Trichoderma* inoculation. In contrast, the expression of the ACO family (*AhACO1*: LOC_02019, *AhACO*: LOC_64097), which has ACO activity and catalyzes the formation of ethylene from pyruvic acid, was significantly downregulated. Moreover, the ACS family (*AhACS3*: LOC_20599, *AhACS1*: LOC_97527) and the GRF family (*AhUGT76F1*: LOC_15453, *AhUGT76B1*: LOC_65278) were also downregulated by *Trichoderma* inoculation (**Figure 6A**).

Under the biotic stress of stem rot disease, significant upregulation by *Trichoderma* inoculation was observed in the IAA family (*AhARG7*: LOC_36955, *AhIAA27*: LOC_020016) and DFR family (*AhSUMM2*: LOC_91374, *AhRSP2*: LOC_96835) in the transcriptomes of peanut seedlings. Conversely, the pectinesterase family (*AhPME31*: LOC_83586, *AhPME53*: LOC_51283, *AhPME2*: LOC_28223, *AhPME53*: LOC_90174), another set of ACO-associated genes (*AhACO1*: LOC_02019, *AhACO*: LOC_64097), and the genes in the PR1 family (*AhPR1*: LOC_14746, *AhBPR1*: LOC_84855), the ERF family (*AhERF2*: LOC_97698, *AhERF*: LOC_89826, *AhERF9*: LOC_37698), and the ACS family (*AhACS3*: LOC_20599, *AhACS1*: LOC_97527) were significantly downregulated (**Figure 6B**).

**Figure 7:**
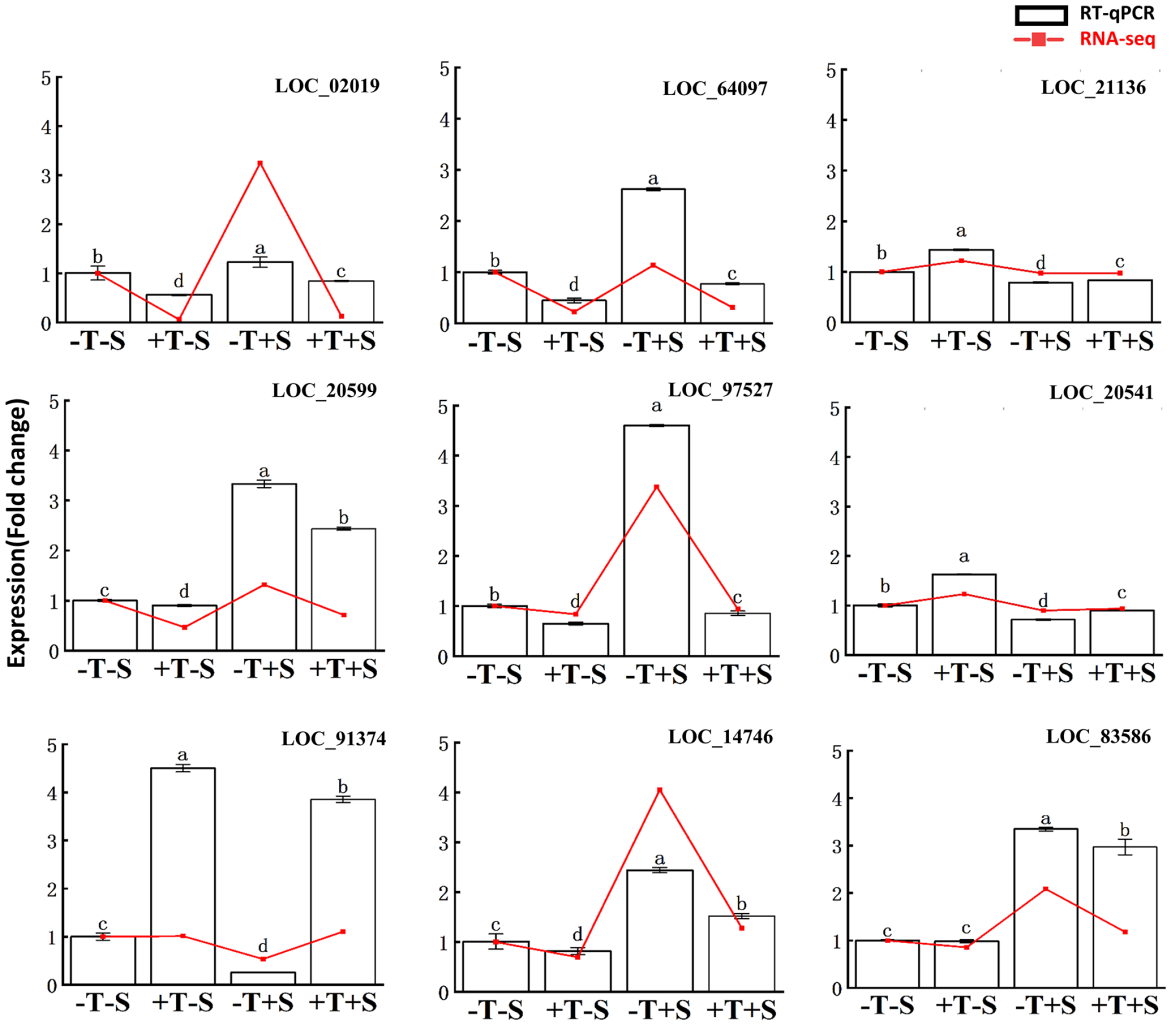
Reverse transcription-quantitative PCR validation of differentially expressed genes in the transcriptomes of peanut root. The relative change of gene expression in two methods was calculated for comparison. The bars represented the average of the biological replicates (*n* = 5) with standard errors. Different letters denote significant differences at *p*< 0.05 by Duncan’s new multiple-range test.

To validate the RNA-seq data, nine DEGs were chosen to quantify the expression level using RT-qPCR (**Figure 7**, **Supplementary Table S2**). The RT-qPCR analysis confirmed that the expression patterns of all nine DEGs were consistent with the RNA-seq data (**Figure 7**). These findings validate the accuracy of the transcriptome data and confirm the association of these genes with the plant growth promotion and disease suppression effects from the strain QT20045.

## 4 Discussion

The study investigates the mechanisms underlying plant growth promotion and disease suppression by *T. harzianum* QT20045 in peanut seedlings. Under conditions without biotic stress, QT20045 significantly enhances root development and height in peanut seedlings by modulating hormone metabolism, particularly increasing IAA content and decreasing ethylene production. This modulation is attributed to the upregulation of genes involved in IAA biosynthesis and downregulation of genes in ethylene biosynthesis, especially both the ACS- and ACO-associated genes. Additionally, QT20045 exhibits antagonistic effects on the pathogen *Sclerotium rolfsii*, employing mechanisms such as entanglement, parasitism, and dissociation. Under the biotic stress of *S. rolfsii*, QT20045 upregulates genes related to pectinesterase, contributing to plant cell wall stability and resistance to pathogens, as well as the genes associated with immune receptors. Overall, *T. harzianum* QT20045 shows potential for enhancing plant growth and suppressing disease in peanut seedlings through hormone modulation, antagonistic activity against pathogens, and enhancement of plant defense mechanisms. The occurrence, prevalence, and severity of plant diseases are influenced by a complex interplay of climate, crop rotation, and agronomic practices (Singh et al., 2023), which can elevate the risks associated with applying (bio)pesticides if the disease does not develop. Our findings indicate that *T. harzianum* QT20045 has both the biofertilizer and biopesticide potential to promote peanut productivity irrespective of stem rot disease occurrence.

### 4.1 Mechanism of plant growth promotion b*y T. harzianum* QT20045

Under the condition with no biotic stress, *T. harzianum* QT20045 considerably enhanced the growth of peanut seedlings by fostering increased root development and height. This enhancement was attributed to its modulation of hormone metabolism within the host plants, including IAA and ethylene. Firstly, *T. harzianum* QT20045 considerably increased the IAA content in the roots of peanut seedlings, possibly because it could activate the expression of the genes related to IAA biosynthesis of the host plants, e.g., the genes involved in IAA biosynthesis *AhIAGLU* were upregulated by QT20045 (**Figures 4**, **7**).

In addition, under no biotic stress, *T. harzianum* QT20045 was able to decrease ethylene production to promote plant growth. Ethylene biosynthesis consists of two primary continuous enzymatic reactions: (i) ACS converts S-adenosylmethionine to ACC and (ii) ACO transforms ACC into ethylene in various plant organs (Bleecker and Kende, 2000). Our study confirmed that *T. harzianum* QT20045 reduced ethylene biosynthesis by regulating both ACS and ACO enzymes. Firstly, the enzyme activity of ACS and ACO was decreased by *Trichoderma* inoculation (**Figures 4C, D**); then, we found that QT20045 suppresses the expression of both ACS-associated genes such as *AhACS3* and *AhACS1* and ACO-associated genes such as *AhACO1* and *AhACO*, while the previous reports only confirmed that *Trichoderma* inoculation enhanced the ACC deaminase activity of the host plant, but not the specific reactions of ethylene biosynthesis (Qi and Zhao, 2013; Zhang et al., 2019).

### 4.2 Mechanism of disease suppression b*y T. harzianum* QT20045

In our study, *T. harzianum* QT20045 exhibited a significant antagonistic effect on *S. rolfsii*, and the pot experiment further confirmed the biocontrol efficacy of *T. harzianum* QT20045 for peanut stem rot disease. The antagonistic mechanisms *Trichoderma* employed included entanglement, parasitism, and dissociation for the mycelia of *S. rolfsii* (**Figure 1**). The results of this study are in accordance with the previous findings that *Trichoderma* species such as *T. harzianum* and *T. longibrachiatum* were able to inhibit the growth of *S. rolfsii* based on *in vitro* tests (Hirpara et al., 2017; Ayyandurai et al., 2023). Interestingly, PR1 gene is considered as an indicator showing a plant’s responses to fungal attack (Anisimova et al., 2021), while our results showed that the application of *Trichoderma* reduced the expression of genes in the PR1 family. This suggests that *Trichoderma*’s presence inhibits the growth of *S. rolfsii* in the soil, consequently weakening the host plant’s immune response.

Under the biotic stress of *S. rolfsii*, inoculation with *T. harzianum* QT20045 significantly improved the growth of peanut seedlings by promoting greater root growth and height while also decreasing disease infection. This improvement was linked to the elevated production of IAA by upregulating the genes of IAA biosynthesis *AhIAGLU* and the reduced release of ethylene by downregulating the genes of *AhACO* in the host plant. Therefore, it is interesting that the presence of peanut pathogens did not affect the plant-growth promoting effect of *Trichoderma* inoculation, even if the same genes were regulated by *T. harzianum* QT20045. Our results indicated the conserved effect of *Trichoderma* on the IAA and ethylene metabolisms of the host plants.

*T. harzianum* QT20045 inoculation leads to the downregulation of the genes in the pectinesterase family of the peanut seedlings, which indicated that *Trichoderma* was able to keep the host plant’s cell wall stable, reduce the degradation of the key cell wall components such as pectin, and enhance the plant’s resistance to pathogens during the infection. Plant cell walls, comprising polymers like cellulose, hemicellulose, pectin, and lignin, are vital not only for maintaining and supporting cells but also for resisting pathogen invasion. Ke et al. (2013) observed that cell-wall-degrading enzymes, including pectinesterase, were significantly upregulated during pathogen infection, leading to a marked reduction in the pectin content of infected plant cells compared to the control. The genes associated with pectinesterase of tomato plants was downregulated by all of the tested *Trichoderma* strains under biotic stress (Lorito et al., 2010), while another study showed that the pectinesterase protein was identified from maize root during the inoculation of *Trichoderma* under hydroponic conditions without pathogens (Guillermo et al., 2018), which indicated that the regulation of pectinesterase genes results from the combined effects of both *Trichoderma* and pathogens. *Trichoderma* inoculation also modulated certain responses of the plant’s immune system—for example, in plant responses to biotic stress, MAPK cascades are crucial for signal transmission, while SUMM2 acts as an immune receptor, responsible for monitoring the integrity of diverse elements within the MAPK cascade (Zhang et al., 2017). Our finding demonstrated that, under the biotic stress of *S. rolfsii*, *T. harzianum* QT20045 was able to upregulate the *AhSUMM2* gene in the peanut seedling but did not affect the MAPK genes.

## 5 Conclusion

Stress from *S. rolfsii* impeded peanut seedlings’ growth, but inoculation with the beneficial microorganism *T. harzianum* QT20045 significantly mitigated this negative impact. QT20045 exhibits beneficial effects on the growth of the host plants by enhancing ACC deaminase activity and IAA production. The key genes sensitive to *Trichoderma* inoculation were identified in the present study, including *AhIAGLU* for IAA metabolisms, *AhACS* and *AhACO* for ethylene modulation, and *AhSUMM2* for enhancing the plant’s resistance to pathogens. These genes can be the focus in future research to investigate the mechanisms by which *T. harzianum* QT20045 regulates the genetic responses of the host plant. The use of *Trichoderma* can significantly diminish the reliance on chemical fertilizers and pesticides, promoting more sustainable and environmentally friendly agricultural practices and securing long-term soil fertility and health.

## Data availability statement

The datasets presented in this study can be found in online repositories. The names of the repository/repositories and accession number(s) can be found in the article/**Supplementary Material**.

## Author contributions

XW: Conceptualization, Data curation, Formal analysis, Investigation, Methodology, Software, Validation, Visualization, Writing – original draft, Writing – review & editing. ZZ: Conceptualization, Data curation, Formal analysis, Validation, Visualization, Writing – original draft, Writing – review & editing. HL: Conceptualization, Investigation, Methodology, Validation, Writing – review & editing. YW: Conceptualization, Formal analysis, Supervision, Validation, Writing – review & editing. JH: Conceptualization, Data curation, Formal analysis, Software, Visualization, Writing – review & editing. HY: Conceptualization, Methodology, Writing – review & editing. YZ: Conceptualization, Formal analysis, Supervision, Validation, Visualization, Writing – review & editing. JL: Conceptualization, Formal analysis, Funding acquisition, Investigation, Project administration, Resources, Supervision, Writing – review & editing.
