# Erratum: The growth-promoting and disease-suppressing mechanisms of *Trichoderma* inoculation on peanut seedlings

**DOI:** 10.3389/fpls.2024.1487731

**Published:** 2024-09-09

**Authors:** Frontiers Production Office

**Affiliations:** Frontiers Media SA, Lausanne, Switzerland

**Keywords:** soil health, microbiome, omics, ACCd, fungal, legume

Due to a production error, reviewer Yunus Korkom was omitted from the article.

The publisher apologizes for this mistake.

The original version of this article has been updated.

