# Supplementary material for: The growth-promoting and disease-suppressing mechanisms of Trichoderma inoculation on peanut seedlings
Source: Front Plant Sci. 2024 Jun 25;15:1414193. doi: 10.3389/fpls.2024.1414193 (PMC11231372; doi:10.3389/fpls.2024.1414193)
Supplement: Supplementary file 1 [file Table_1.docx]

Supplementary Material

# Supplementary Data

## 1.1 Supplementary Tables

#### **Supplementary Table S1.** Biocontrol assay of *T. harzianum* QT20045 against peanut stem rot disease in the greenhouse.

| **treatments** | **Incidence (%)** | **Disease index (%)** | **Control efficiency (%)** |
| --- | --- | --- | --- |
| -T-S | 0.00±0.00 | 0.00±0.00 | - |
| +T-S | 0.00±0.00 | 0.00±0.00 | - |
| -T+S | 61.26±4.88 a | 49.95±3.66 a | - |
| +T+S | 26.67±6.67 b | 35.47±3.33 b | 68.79±3.33 |

**Notes:** Values are the means ± standard error. Different letters in the same column mean significant difference at the p < 0.05 level by Duncan’s new multiple range test.

**Supplementary Table S2.** The detailed information of the fifteen DEGs and primer pairs for RT-qPCR.

| **Gene name** | **Gene Description** | **Primer sequences（5’→3’）** | **product length** |
| --- | --- | --- | --- |
| LOC_02019 | 1-aminocyclopropane-1-carboxylate oxidase 1 | F：TGCAGGTGGCATCATTCTGT  R：GACCCTGTGCACAACACTCT | 175bp |
| LOC_64097 | 1-aminocyclopropane-1-carboxylate oxidase | F：GCTAACTACCCTCCATGCCC  R：TATCACACGGTGCTCCACAC | 234bp |
| LOC_83586 | pectinesterase 31 | F：ATCACTCTTGCTGGCCTCAC  R：CTAATTGCCACTGCTTGCCC | 209bp |
| LOC_91374 | SUMM2; NB-LRR protein SUMM2 | F：GTGGGTGTGGATTCGATGGT  R：TCGTCCTTCACCCCTAACCT | 239bp |
| LOC_51283 | pectinesterase 53 | F：GTCCCTTTTCTCGCGTGGTA  R：AAGGCTTGGCTTCATCGTCA | 195bp |
| LOC_20599 | 1-aminocyclopropane-1-carboxylate synthase 3 | F：TGGTGTCTTCCCAAACCCAG  R：CCCAACAGAACAACCCAGCA | 177bp |
| LOC_97527 | 1-aminocyclopropane-1-carboxylate synthase 1 | F：GAGCCTGGTTGGTTTAGGGT  R：CAAGGGGTGAGTGAGGCATT | 229bp |
| LOC_21136 | IAGLU1; UDP-glucose:(indol-3-yl)acetate beta-D-glucosyltransferase | F：TCAAAGGGTGTCCACGTCAC  R：AGCTTCGATCGTGCTCCTTT | 241bp |
| LOC_20541 | IAGLU2; UDP-glucose:(indol-3-yl)acetate beta-D-glucosyltransferase | F：ACTGGTCAGATCAACCGACG  R：ACGAACCACCATCCACCATC | 219bp |
| LOC_14746 | PR1; pathogenesis-related protein 1 | F：ACGTGGCCTATGCTCAAGAC  R：TGCTTTTCGTCCACCCACAA | 247bp |
| LOC_83668 | SGT1；UDP-glycosyltransferase 74G1 | F：TTTGACCACACTGGCCCTTC  R：AAAGCAGCACCAACAATCCC | 200bp |
| LOC_97698 | ERF2; ethylene-responsive transcription factor 2 | F：CGTTATAGGGGTGTGAGGCG  R：TCCGTTTTACAGCGGATGCT | 247bp |
| LOC_37698 | ERF9; ethylene-responsive transcription factor 9 | F： TGAGTCATCAACACCGGAGC  R： GCCACCACACCATCAACAAC | 165bp |
| LOC_84604 | actin-7 | F：TTGAGACGGCAAAGAGCAGT  R：CCAGGGAACATGGTAGACCC | 244bp |

**Supplementary Table S3.** The CAS/Products number of the chemicals used in the experiment.

| **Chemicals**  **name** | **CAS/Products number** |
| --- | --- |
| Indole-3-acetic acid（IAA） | NO.87-51-4 |
| 1-aminocyclopropane-1-carboxylate acid (ACC) | NO.22059-21-8 |
| 2,4-dinitrophenylhydrazine | NO.119-26-6 |
| Bovine serum albumin | NO.9048-46-8 |
| Methanol | NO.67-56-1 |
| Acetonitrile | NO.67-56-1 |
| Acetic acid | NO.64-19-7 |
| Ethylene | NO.74-85-1 |
| HEPPS-KOH | NO.16052-06-05 |
| Pyridoxal phosphate | NO.54-47-7 |
| β-sulfhydryl ethanol | NO.60-24-2 |
| S-Adenosyl-L-methionine (SAM) | NO.3493-13-8 |
| MOPS | NO.1132-61-2 |
| Ascorbic acid | NO.[50-81-7](https://www.sigmaaldrich.com/US/en/search/50-81-7?focus=products&page=1&perpage=30&sort=relevance&term=50-81-7&type=cas_number) |
| NaHCO_3_ | NO.144-55-8 |
| FeSO_4_ | NO.7782-63-0 |
| Strand Synthesis Reaction Buffer | NO.9068-38-6 |
| M-MuLV Reverse Transcriptase(RNase H-) | NO.9068-38-6 |
| DNA Polymerase I | NO.[9012-90-2](https://www.sigmaaldrich.com/US/en/search/9012-90-2?focus=products&page=1&perpage=30&sort=relevance&term=9012-90-2&type=cas_number) |
| Potato Dextrose Agar (PDA) | NO.[P2182](https://www.sigmaaldrich.com/US/en/product/sial/p2182) |
| Potato Dextrose Broth (PDB) | NO.P6685 |

**Notes:** Chemical samples were obtained from Sigma-Aldrich china.
